# Eating disorder in males children and adolescents: a case series

**DOI:** 10.1192/j.eurpsy.2026.11381

**Published:** 2026-07-31

**Authors:** R. Arias Hidalgo, C. Canga Espina

**Affiliations:** 1 Hospital Puerta de Hierro, Madrid; 2 Clinica Universidad de Navarra, Pamplona, Spain

## Abstract

**Introduction:**

Eating disorders (ED) are psychiatric diseases associated with significant morbidity and mortality with a greater prevalence in the female gender. This has led to the presence of little literature on eating disorders in men, so that there is limited understanding and non-specific orientation of the disorder in this population. We present a series of five clinical cases of ED diagnosis in male children who attended our Child and Adolescent Psychiatry Unit during the last years.

**Objectives:**

Descriptive study.

**Methods:**

- **Case 1:** 15-year-old male developed restriction and purging after peer comments, leading to severe malnutrition (BMI 14.5), depression, and bradycardia. Improved with hospitalization, enteral nutrition, pharmacotherapy, and family therapy; body image concerns and excessive exercise persisted.
- **Case 2:** 12-year-old boy with anxiety and obsessive-compulsive traits presented with restriction, excessive exercise, and muscularity concerns (BMI 15.3). Treated with sertraline, psychotherapy, and nutrition; gradual improvement, though low BMI and muscle-focused goals persisted.
- **Case 3:** 14-year-old male with ADHD developed selective eating and weight loss (BMI 17.3) without body image concerns. Sertraline and nutritional counseling led to weight recovery and social/academic improvement over 16 months.
- **Case 4:** 9-year-old boy developed restriction and excessive exercise after peer comments and maternal illness (BMI 15.3), with bradycardia and hypoglycemia. Recovered with pharmacotherapy, psychotherapy, and intensive follow-up after relapse; fear of weight gain remained.
- **Case 5:** 11-year-old boy with body dissatisfaction and paternal modeling developed restriction and exercise, severe malnutrition (BMI 13.4), depression, and suicidal thoughts. Required two hospitalizations with enteral nutrition, pharmacotherapy, and exposure-based feeding therapy; gradual improvement achieved.

**Results:**

These cases highlight the variability of eating disorder presentations in male children and adolescents, including restrictive eating, selective intake, and muscularity-focused concerns. Early warning signs such as compulsive exercise, growth stagnation, and psychosocial distress may precede classical symptoms. Family involvement and gender-sensitive approaches are essential for early detection, tailored intervention, and improved outcomes.

**Image 1:**
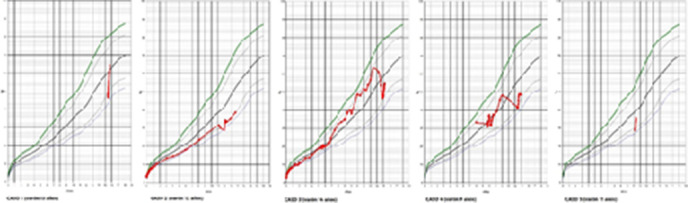
Image 1: Long description.

**Image 2:**
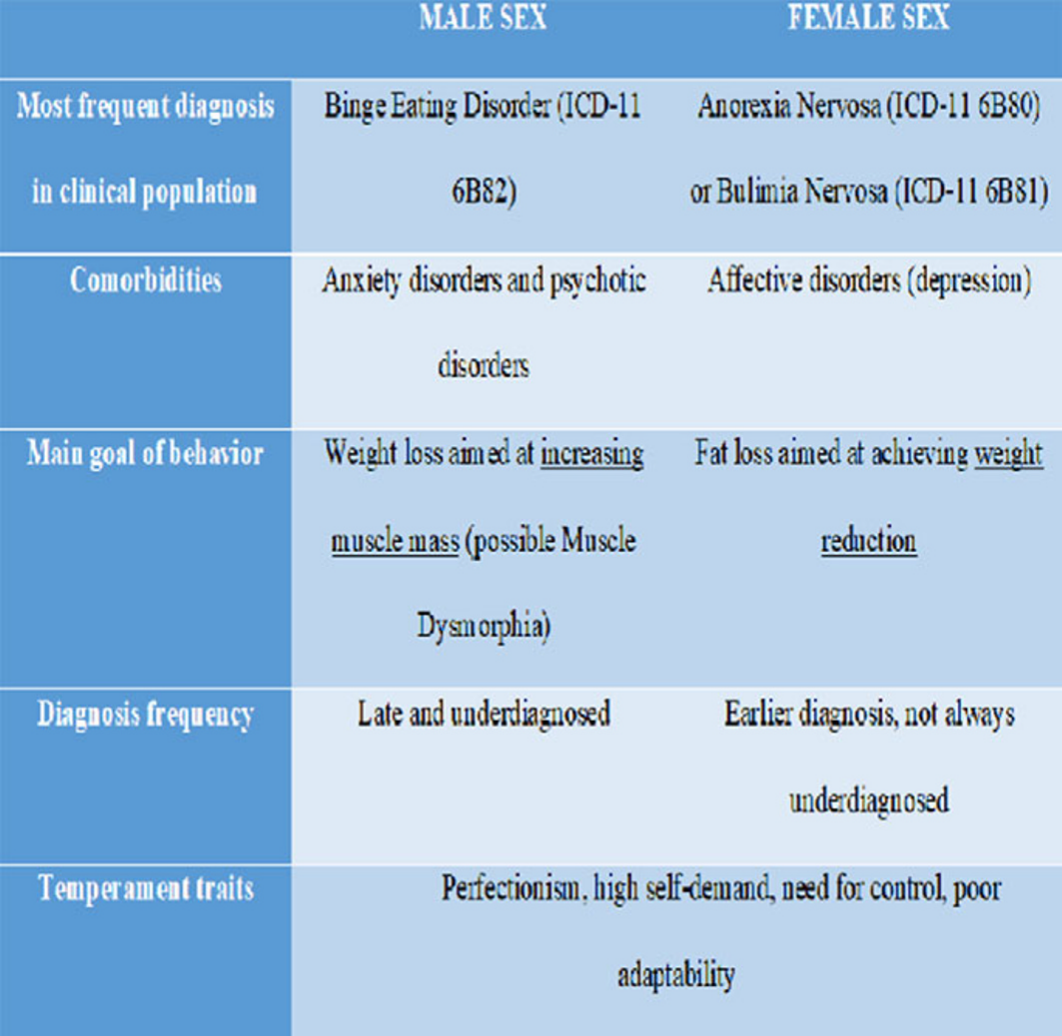
Image 2: Long description.

**Conclusions:**

Eating disorders in males are underdiagnosed, but incidence is rising; pediatricians need training for early detection.

Most common EDs: Binge Eating Disorder, Bulimia, and Anorexia; anxiety is the most frequent comorbidity.

Males often aim to reduce fat and increase muscle; gender-sensitive diagnostic tools are required.

Early warning signs: compulsive or altered exercise patterns; purging behaviors are less common.

Boys lack clear biological markers (e.g., amenorrhea); growth stagnation is a critical red flag.

Further research is needed to develop male-specific diagnostic tools and treatment approaches.

**Disclosure of Interest:**

None Declared

## Long descriptions

### Image 1: Long description

A five-panel series of line graphs. Each graph shares a common background of reference growth curves: a top green curve, a middle black curve, and two lower light blue curves. The x-axis represents time in days and the y-axis represents a physiological measurement.

* Panel 1 (Case 1): A red data line follows the lower blue reference curves until day 15, where it shows a sharp vertical spike toward the black curve.

* Panel 2 (Case 2): The red line begins at the bottom left and follows the lowest blue curve closely with minor fluctuations through day 20.

* Panel 3 (Case 3): The red line shows a steady upward trend, crossing from the blue curves to the black curve, peaking near day 15 before a sharp downward and then upward jagged movement.

* Panel 4 (Case 4): The red line starts mid-graph around day 7, fluctuating horizontally between the blue and black curves before a slight upward trend toward day 18.

* Panel 5 (Case 5): The red line is only visible as a short, nearly vertical segment between days 12 and 13, positioned between the blue and black reference curves.

Navigate back to Image 1:.

### Image 2: Long description

The table consists of three columns. The first column lists categories, the second column is titled MALE SEX, and the third column is titled FEMALE SEX.

* Most frequent diagnosis in clinical population: For males, Binge Eating Disorder (I C D-11 6B82). For females, Anorexia Nervosa (I C D-11 6B80) or Bulimia Nervosa (I C D-11 6B81).

* Comorbidities: For males, Anxiety disorders and psychotic disorders. For females, Affective disorders (depression).

* Main goal of behavior: For males, Weight loss aimed at increasing muscle mass (possible Muscle Dysmorphia). For females, Fat loss aimed at achieving weight reduction.

* Diagnosis frequency: For males, Late and underdiagnosed. For females, Earlier diagnosis, not always underdiagnosed.

* Temperament traits: This row spans both sex columns and lists Perfectionism, high self-demand, need for control, and poor adaptability.

Navigate back to Image 2:.

